# Supplementary material for: Splice-Junction-Based Mapping of Alternative Isoforms in the Human Proteome
Source: Cell Rep. Author manuscript; Available in PMC 2020 Jan 15. (PMC6961840; doi:10.1016/j.celrep.2019.11.026)
Supplement: 2 [file NIHMS1546469-supplement-2.zip › DF1/PXD010154/Ovary/Ovary_5_ARHGEF12_LQDGAVVTPSRPLGDTLTVSEAETDPGDVLGR.pdf]

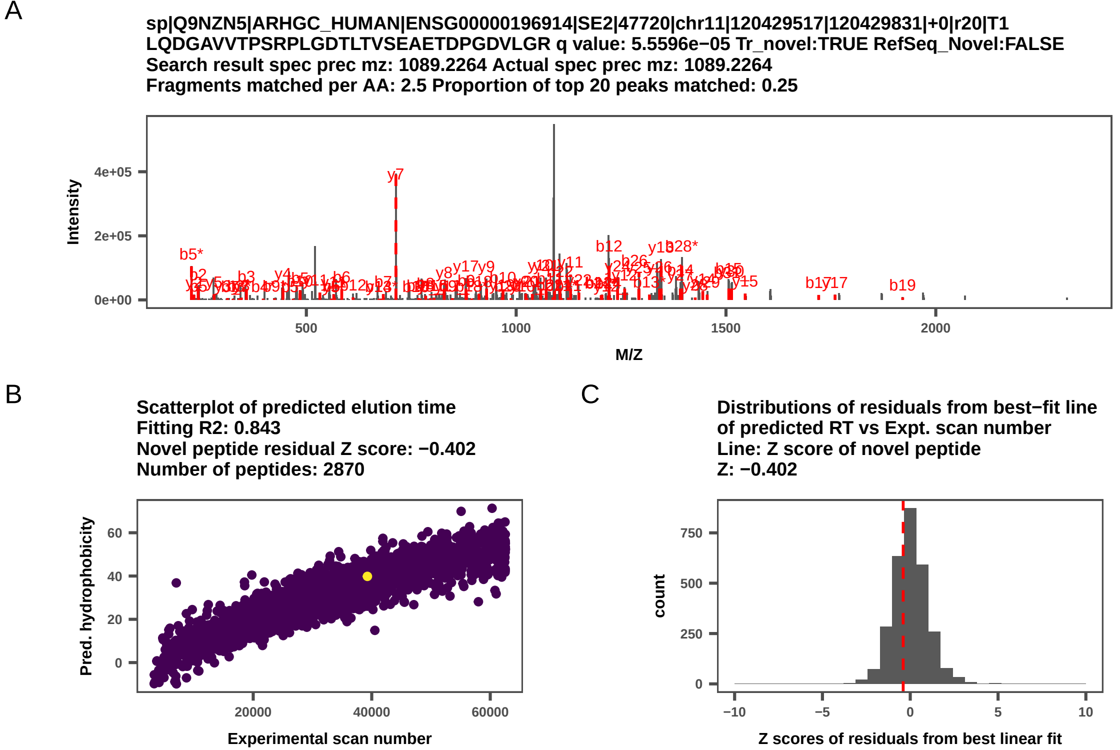
A
sp|Q9NZN5|ARHGC_HUMAN|ENSG00000196914|SE2|47720|chr11|120429517|120429831|+0|r20|T1
LQDGAVVTPSRPLGDTLTVSEAETDPGDVLGR q value: 5.5596e−05 Tr_novel:TRUE RefSeq_Novel:FALSE
Search result spec prec mz: 1089.2264 Actual spec prec mz: 1089.2264
Fragments matched per AA: 2.5 Proportion of top 20 peaks matched: 0.25
Intensity
4e+05
2e+05
0e+00
y7
b5*
b12
b28*
b19
y15
M/Z
500
1000
1500
2000
B
Scatterplot of predicted elution time
Fitting R2: 0.843
Novel peptide residual Z score: −0.402
Number of peptides: 2870
Pred. hydrophobicity
60
40
20
0
20000
40000
60000
Experimental scan number
C
Distributions of residuals from best−fit line
of predicted RT vs Expt. scan number
Line: Z score of novel peptide
Z: −0.402
count
750
500
250
0
−10
−5
0
5
10
Z scores of residuals from best linear fit
